# Posterior Lamellar Graft Preparation: A Prospective Review from an Eye Bank on Current and Future Aspects

**DOI:** 10.1155/2013/769860

**Published:** 2013-05-30

**Authors:** Mohit Parekh, Gianni Salvalaio, Alessandro Ruzza, Davide Camposampiero, Carlo Griffoni, Alfonso Zampini, Diego Ponzin, Stefano Ferrari

**Affiliations:** ^1^The Veneto Eye Bank Foundation, Padiglione Rama, Via Paccagnella 11, Zelarino, 30174 Venice, Italy; ^2^Department of Ophthalmology, Dell'Angelo Hospital, Zelarino, 30174 Venice, Italy

## Abstract

Descemet membrane endothelial keratoplasty (DMEK) is a corneal surgical technique which selectively replaces the damaged posterior part of the cornea with a healthy donor graft retaining the rest of the tissue intact. There is a need to validate and standardize the donor tissue before grafting due to certain issues that can lead to consequences such as graft failure due to poor endothelial cell count, higher mortality, detachment of the graft, or increased surgical expenses, time, and effort. Thus, prospective potential surgeons and eye banks should now aim at developing new improved surgical techniques in order to prepare the best suited, validated, precut, preloaded, and easy to transplant tissue to reduce pre- and postsurgical complications. This could be achieved by defining parameters like graft thickness, accepted mortality threshold of the endothelial cells, and behavior of grafts during preservation and transportation along with using more sophisticated instruments like microkeratome and femtosecond lasers for graft preparation. Thus, a rapport between the eye banks and the surgeons along with the advanced instruments can overcome this challenge to find the best possible solution for endothelial keratoplasty (EK).

## 1. Introduction

**Figure 1:**
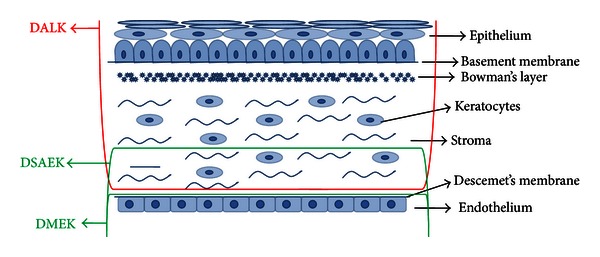
Different layers of a corneal tissue and types of surgeries that involve specific layers. DALK replaces the anterior part, whereas DMEK and DSAEK replace the posterior part of the cornea.

The cornea is the anterior part of the eye globe. It is an avascular tissue which is directly exposed to the external environment. It focuses the image by refracting the light to the retina through a lens. Hence, it should be clear and well maintained for optimal visibility [1]. The cornea acts as a shield against external dust or microbes and prevents them to enter the eye globe. Damage or disturbance to the cornea due to scar, foreign bodies, or other diseases or disorders can lead to poor visibility. Cornea is made up of six layers which are responsible for the organization of the corneal cellular matrix which in turn is important for guiding the light to the retina. The corneal layers are the epithelium, basement membrane, Bowman's layer, stroma, Descemet's membrane, and endothelium (Figure 1) [1]. Each layer has its own specificity, but when corneal transplantation especially penetrating keratoplasty (PK)/endothelial keratoplasty (EK) is considered, the endothelium has a more important role to play as it does not have the capacity to regenerate and hence should be left viable and undisturbed. Endothelial damage or poor viable cell count is assumed to be majorly responsible for graft rejection [2].

Corneal lamellar keratoplasty (LK) is a surgical technique that allows preserving healthy portions of the cornea while selectively replacing the dysfunctional segments. The best action to treat corneal disorder is a replacement of the damaged recipient cornea (complete/partial) with a healthy donor corneal tissue. Deep anterior lamellar keratoplasty (DALK) is a surgical technique that is considered the best for treating the patients with anterior layer (epithelium, Bowman's layer, and stroma) disorders. Descemet's membrane endothelial keratoplasty (DMEK) is currently pursued to treat the patients with endothelial dysfunction. With time, Descemet's stripping automated endothelial keratoplasty (DSAEK) has evolved drastically with an instrument such as a microkeratome, which is more standardized, efficient, and safe to create corneal grafts or lenticules. The different techniques for specific layers are illustrated in Figure 1. New tools and advanced machineries like ultrasonic pachymetry, microkeratomes, excimer laser, and more recently femtosecond (FS) lasers have enhanced the ability to work with more safety and accuracy in tedious microsurgical environments. Viscoelastics and artificial chambers have proved beneficial to maintain the cellular viability. The above mentioned surgeries have enlarged the view of corneal surgery by achieving higher visual outcomes as compared to PK while limiting the rate of rejection and increasing the long-term graft stability. Further research is showing promising results with EK using thinner tissues and an expected long-term visual outcomes and graft stability [3–5].

## 2. Anterior Lamellar Keratoplasty (ALK)

ALK targets the replacement of the damaged anterior segment (epithelium and part of stroma) of the recipient's cornea with the anterior part of the healthy donor tissue. The deeper layers (posterior) of the recipient cornea, specifically the endothelium and the Descemet's membrane, are left intact which reduces the risk of rejection and therefore has a distinct advantage over PK. Deep anterior lamellar keratoplasty (DALK) replaces both, the epithelium and most of the stroma with the donor tissue. This is favorable for those disorders which affect the anterior segment of the cornea [6–9]. According to the literature, with new instruments, DALK has now shown equal results as PK, if not better than PK, that are also based on best spectacle corrected visual acuity (BSCVA) [6, 10]. Smoothness of the stromal interface is ultimately related to better visual outcome; however, scarring of the stroma is still an issue with DALK. It has been found that instruments like FS lasers have been successfully used to cut the lamellar flaps, and it is believed that they could also be used for DALK in order to reduce the irregular scarring. Thus, with less compromise to the endothelium, DALK is considered as the primary choice of treatment for most anterior corneal disorders [10].

## 3. Endothelial Keratoplasty (EK)

EK selectively replaces the diseased corneal endothelium with healthy donor tissue through a small limbal incision while retaining the healthy anterior part of the patient's cornea. This surgical technique has multiple advantages over PK as the recipient cornea remains structurally intact and resistant to injury. In addition, since it is a suture-less surgery, the results lead to quick rehabilitation and better visual outcomes. In general, the recipient eye is maintained much stronger as compared to PK. The different types of EK currently pursued are the following. 

### 3.1. Deep Lamellar Endothelial Keratoplasty (DLEK) or Posterior Lamellar Keratoplasty (PLK)

This procedure is the first hand/primary technique with excision of the posterior recipient stroma and endothelium with small curved scissors and trephine. The donor tissue is folded for insertion through a small incision near the limbus region. 

### 3.2. Descemet's Membrane Endothelial Keratoplasty (DMEK)

 The posterior membrane which includes the Descemet's membrane and the endothelial cells is excised and transplanted. The thinnest possible lamellar graft is transplanted with the intention of better and faster visual recovery and outcomes. Majority of the tissues are prepared today with an air bubble technique. The cellular mortality after the preparation of the graft can be a major concern if the DMEK is created by stripping it off manually. 

#### 3.2.1. Recent Advances in DMEK

A recent study described by Dapena et al. showed a no-touch technique for DMEK surgery. As per this study, the technique could provide best corrected visual acuity (BCVA) of 20/25 or more with a good endothelial cell density after 6 months from surgery. The steps include incision and descemetorhexis (excision of Descemet's membrane from the recipient cornea), preparation and implantation of DMEK graft followed by orienting, unfolding, centering, positioning, and fixing the DMEK graft. This technique claims to be more standardized with near complete visual recovery and minimal endothelial cell loss. It further explains that approximately 95%of the cases may gain a BCVA of 20/40 or better and 75% may attain 20/25 within 6 months post-op [11]. 

Another report cited a combination of two procedures, that is, preparation of “no-touch” DALK and DMEK grafts from the same donor. The rolled tissue was placed on a soft contact lens which was used for trephination of the endothelial graft using a custom-made trephination system. This technique claims to produce undamaged grafts with better handling of the tissues especially for thin Descemet's membrane. As the undamaged anterior cornea could also be used after separating the Descemet's membrane, this method increases the availability of the donor tissue by using two different grafts from the same donor. No clinical signs of graft dysfunction, primary/secondary graft failures, or graft detachments were observed. The endothelial cell density and the relative mortality showed no significance in terms of cell loss before and after technique [12].

Descemet endothelial graft (DEG) can be isolated as in the studies described above or isolated after pneumatic dissection (inserting pressurized gas for creating mechanical motion) using air bubble technique and preserving the lenticules for 7 days in organ culture. In the latter studies, the anterior stroma was removed using the microkeratome followed by air injection to separate the Descemet's membrane and the stroma and then attached to a silicone weight using a scleral ring. This technique showed that the DMEK tissues can be pre-prepared in the eye banks and can be preserved with a minimum endothelial cell loss [13]. 

However, a similar study showed that although using air as a medium to create the bubble could be useful, the lenticule demonstrated the presence of residual stroma in all the tissues that were harvested (*n* = 5; average stromal residue = 12.45 micrometers). This concludes that although with a possibility of creating a thin lenticule, the presence of stroma indicates that the technique should not be termed as DMEK but a very thin DSEK [14].

Similarly, microkeratome and Barraquer sweep assisted lamellar preparation was another technique for harvesting donor DM and endothelium which also showed minimal stromal interference with higher endothelial cell integrity and minimal cell loss. The anterior stroma was removed using Moria One microkeratome, whereas the residual stromal bed over the central cornea was removed by blunt dissection using a Barraquer sweep. This technique explains that a thin rim of posterior residual stroma permits easy donor button trephination and tissue manipulation. Optical coherence tomography evaluation revealed minimal stroma underlying DM and endothelium [15]. This technique also shows the presence of residual stroma; therefore, although with a very thin lenticule preparation, could this technique be termed as DMEK?

A few other studies include creating endothelial lenticule using DMEK-S technique. A corneoscleral disc was mounted on a barron artificial anterior chamber with endothelial side facing up. A big bubble technique was used to separate the DM and the stroma. The disc was turned over and approximately 80% of the stromal tissue was removed. The central stroma (6 mm diameter) was marked with letter “S” and the rest of the stroma was amputated. This technique showed less endothelial cell loss and claimed to be a potential method as it required no special surgical instruments. Although the endothelial cell density was noted, the mortality after the lenticule preparation was not recorded. The ECD 1 year post-op showed approximately 44% loss. Therefore, the hypothesis could be (a) the cell loss was due to the recipient acclimatization post-op or (b) the transplanted cells were damaged or dead after graft preparation. The mortality checks therefore become an important parameter and an issue that needs to be highlighted for the DMEK surgeries. Hence, determining the mortality after graft preparation is recommended [16]. 

In conclusion, there are several methods that have been introduced which have different approaches to retrieve the DEG, preserve and supply as either precut tissue from the eye banks or preprepare at the surgical theatre. However, there is a lack of a standardized method which can repeatedly prove the reduction of risk or complications that are usually seen due to unidentified parameters like mortality and other risks or complications such as graft failure due to detachment or poor endothelial cell count post-op. 

**Figure 2:**
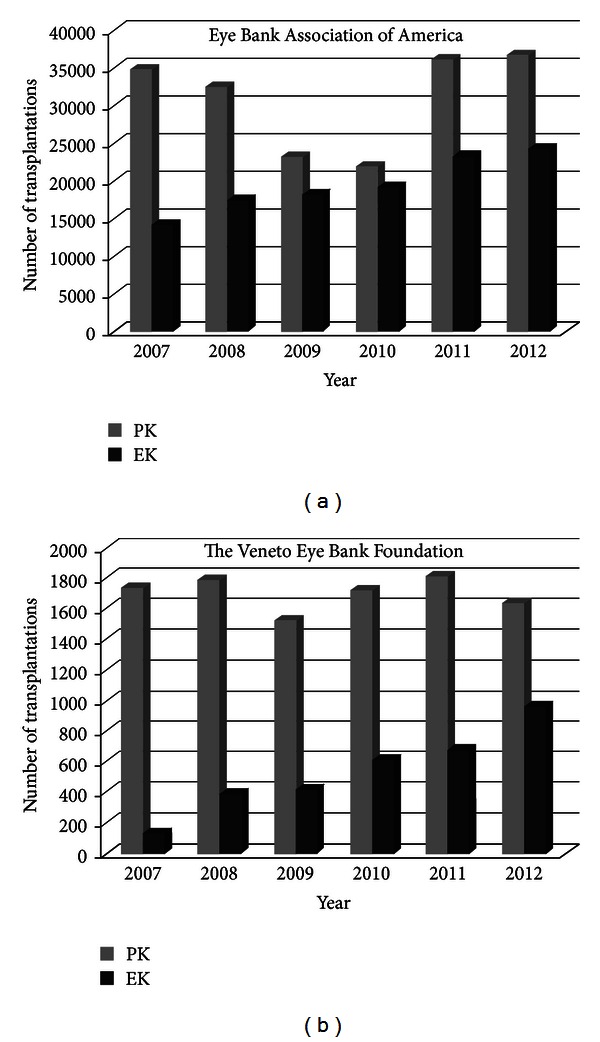
The data shows the increasing number of EK transplants per year in the (a) United States of America (eye banking statistical report, Eye Bank Association of America, 2012) and at (b) the Veneto Eye Bank Foundation, Italy (2012 annual statistical report of donation and transplantation).

Even with its limitations, EK has succeeded PK as the first choice of treatment for endothelial dysfunction due to its advantages like quick and efficient surgery with reduced manipulation, low surgical risk, and better visual outcome. Innovations in EK with modification in donor preparations have broadened its use and improved intraoperative ease, and reduced postoperative complications have therefore been responsible for its emerging popularity [17] as shown in Figure 2.

### 3.3. Descemet-Stripping Automated Endothelial Keratoplasty (DSAEK)

It is less traumatic as compared to manual procedures that include scissors and trephines. A mechanical microkeratome is used to simplify the donor tissue dissection, thus, making the procedure more standardized and easy with lesser damages to the prepared graft. Furthermore, as the anterior corneal surface is not manipulated, it does not result in any of those refractive errors that is usually seen after PK. However, it might show a slight hyperopic shift due to changes in the curvature or astigmatism. More recently, FS lasers have also been used for donor tissue dissection [10]. This method includes a little stromal interference and therefore is not a specific DEG preparation-based technique.

## 4. Recent Advances and Future Goals

### 4.1. Use of Femtosecond Laser for Lamellar Keratoplasty

Where Laser-Assisted In Situ Keratomileusis (LASIK) helps to correct the refractive errors, improved FS laser engines are proving to be useful for lamellar and cataract surgeries [18]. Earlier settings for LK used microkeratome which is less expensive, standardized, and provided smoother surfaces for lenticules. However, microkeratome had certain limitations related to poor depth adjustments, poor thickness reproducibility due to microkeratome head sizes, and irregularity of the lenticule interface. FS lasers reduced the complication rate due to flap creation and improved the predictability of flap dimensions and quality of the optical surfaces as compared to the flaps that were obtained by microkeratome [19–24]. 

Other advantages of the FS laser include (a) precise cuts at specific sites; (b) higher reproducibility; (c) reduced dissection issues; (d) standardized procedures with specific thickness; (e) establishing safe and reliable procedure due to satisfactory outcomes with smoother stromal surfaces which is important for long-term visual outcomes [25]. Both safety and reliability of corneal lamellar cuts using IntraLase FS laser (iFS) have been demonstrated extensively for LASIK and recently for EK [25]. Lenticules created with iFS are more planar shaped and thinner, which is essential for better visual outcomes. Nevertheless, the smoothness and regularity of the stromal interface could still be improved [25]. iFS laser uses pulses to create corneal resection. The quality of the surfaces obtained is determined by programmable parameters like the laser spot and line separation and the energy delivered per pulse [19]. The new FS laser machines are well equipped with higher engine speed and closer spot and line separation to create smoother cuts. As described earlier, studies have reported the use of FS lasers to create donor tissues for EK [26–29], whereas others showed better results with ALK [30, 31].

Rousseau et al. showed that the issues arise with the donor corneas when determining the optimal amount of energy for a lamellar cut. The optimal setting should be enough to penetrate deep into the posterior stroma, overcome diffraction, and prevent any keratocyte activation or inflammatory reactions [25]. Posterior collagen lamellae are less interweaved and distributed systematically which impairs the regularity of lamellar cuts performed in the posterior stroma [32]. As the corneal anatomy becomes more compact as we go more posterior, cuts made with laser below 220 *μ*m become more rough and irregular due to reduced laser beam focus. It is believed that setting the spots closer together with more focal energy and adjusting the spot and line separation can help to create a smoother dissection even while creating deeper cuts [32].

iFS laser seems advantageous when transplants involve thin button of anterior corneal tissues. The interface gets rough when the laser reaches deep towards the corneal stroma for full lamellar cut [33]. It has already been studied that iFS lasers can create endothelial lenticules with a good quality of stromal interface which is comparable to refractive surgery [34]. The use of the FS laser to perform lamellar keratoplasty was thus evaluated in several in vitro and animal models [35, 36]. In 2007, Cheng et al. reported the first FS laser-assisted endothelial keratoplasty by preparing the donor cornea using the FS laser [26]. 

The 60 kHz FS laser allows closer spot and line separation with lower energy levels and results in smooth stromal interface also in deeper cuts which states that higher frequency with lower energy and closer spot and line separation can create smoother stromal bed surfaces [19, 20]. Bethke noted that enhancements to 150 kHz FS laser can show better outcomes considering important features like (a) speed, (b) flap creation, and (c) angle variation. It is observed that increasing speed helps to place the laser ablations close together individually, simplifies flap lifts, and smoothens the surface with an overall faster procedure. The speed allows the user to place the laser ablations closer together individually and row by row. Thus, increased laser speed of 150 kHz over 60 kHz allows the surgeon to perform the procedure in a shorter period of time with a tighter spot and line separation. Microkeratome is a manual procedure and therefore standardization is less feasible as compared to FS lasers which are software-based programs [37]. Therefore, FS lasers with higher engine speed and closer spot and line separation units can be a good rescue for preparing the donor grafts for DMEK. 

### 4.2. Ultrathin (UT) DSAEK

DSAEK is a standardized method to perform EK, unlike DMEK. As it reduces the risk of complications and allows a better and faster recovery and visual outcomes, it has become a goldstandard amongst the eye bankers and surgeons. Although good results have been achieved against PK, many surgeons have speculated that DSAEK can perform even better in terms of visual acuity. However, the reason for poor performance is mostly based on a hypothesis related to the presence of a stromal interface. Therefore, the next challenge was to completely remove the stromal interface and create a DEG. 

In 2006, Melles introduced DMEK procedure where only the donor's DEG was stripped off, thus, removing most of the stromal interface. This procedure reported a number of patients with 20/20 or better, but did not exceed 40%. It was therefore concluded primarily that the stromal interference during the EK could not be the only reason of poor visual outcome post-op. Moreover, DMEK requires high surgical skills, tissue preparation followed by surgical time, unlike DSAEK. In addition, a high rate of tissue loss and detachment rate with a huge amount of graft failures have discouraged most surgeons to adopt this technique [2, 4]. Another argument for the graft failure or high rejection rate could be the presurgery mortality checks. Again, the majority of surgeons who prepare the grafts before surgery do not check the endothelial mortality after preparation of the graft and therefore it is difficult to determine the accuracy of the procedure, viability of the transplanted graft and endothelial cell density post-op. This creates a sense of confusion leading to a disturbed conclusion. Therefore, we highly recommend taking this point into consideration as the surgery success depends not only on the acceptance of the graft but also on the recovery and long-term visual outcomes which is also based on viable endothelial cells. 

Recent studies have demonstrated that DSAEK grafts that are thinner than 131 micrometers have shown 20/20 vision post-op. Moreover, hyperopization is reduced due to peripheral edges [5, 38]. DSAEK graft thickness has not been validated or standardized at various places and therefore, it is very difficult to relate the visual outcomes to DSAEK graft thickness. To reduce this complication, a new approach to the conventional DSAEK surgery was introduced in 2009 and named ultrathin (UT) DSAEK by Busin [39]. The preparation procedure, manipulation, and the transportation of the grafts have completely been customized. UT-DSAEK uses a modified conventional microkeratome, which can cut the cornea twice. The first cut is to debulk the donor tissue and the second one to cut the final thickness up to 100 micrometers. This procedure claims to reproduce results with optimal smoothness of the stromal interface and thickness. Other benefits include creating a thin tissue which reduces the wastage of donor tissue significantly [39].

The microkeratome-assisted UT DSAEK preparation showed that double cuts can create lenticules with <100 *μ*m of thickness. The endothelial cell density before and after preparation showed an average loss of approximately 3-4%, although the difference was not found significant. If it is proved that the residual stroma does not interfere with the visual outcome post-op, UT DSAEK could be the future due to its benefits that include standardization, lesser manipulation, or manual error [40].

## 5. Discussion

With the current studies, DMEK, which makes use only of the Descemet's membrane and the endothelium for transplantation, is not a standardized procedure and due to the requirement of high surgical skills only a small number of surgeons are capable of performing it. Moreover, the major drawbacks include the preparation, manipulation of donor material, unpredictable complications, and graft failure rates. Although it has a <40% success rate, up to 16% of graft failure before surgery, approximately 63% of cases with detachments and 8% with primary graft failure [38], it is still used by some surgeons due to lack of a better option. Mortality of the cells is another crucial issue in DMEK, but many surgeons prefer to eliminate the mortality checks once the tissue has been prepared as they mainly target post-op visual outcomes. DSAEK, an alternative surgical option for corneal endothelial disorder, has shown better postoperative results. Ultrathin DSAEK can cut the tissue to the minimum by removing the majority of the stroma (depending on corneal thickness) using 2 cuts which is not the case in conventional DSAEK. Some studies have shown that the best visual outcome can be influenced by graft thickness. According to the hypothesis, if the endothelium is left untouched, then minimum manipulation will result in reduced mortality. Ease of transportation and quick surgery to reduce the overall expenses should be the next goal.

As we speculate, the eye banks will play an important role in the near future for the development of new surgical techniques in collaboration with the clinicians. One of the issues for eye bankers today is that there is no standard or threshold limit for the requirement of the viable endothelial cell count for critical surgeries like DMEK. This creates a lot of confusion, when a surgeon demands a precut tissue for DMEK or when a surgeon is preparing the graft before surgery. Moreover, as mentioned earlier, the majority of surgeons do not calculate the mortality or the endothelial cell density after the tissue preparation, which addresses a challenge to the eye bankers to standardize the mortality issue. This also results in a false positive post-op endothelial cell survival study although the results for surgical success and visual recovery are documented to be positive. 

Thus, in general, the possible future challenges and the key issues which need to be identified and demonstrated for the standardization of the EK procedures could be the following.

1. Thickness: can thinning the tissues to the minimum (DEG) be really useful in terms of visual outcome?
2. Viable endothelial cell density: should the surgeons evaluate the mortality or the viable endothelial cell density after DMEK graft preparations before surgery?
3. Standardization: can a standardized and validated pre-cut, preloaded tissue help to reduce the risk, time, and cost of the surgery?

Thinning the tissues using UT DSAEK has provided better results, but needs to undergo a strong confirmation to practically prove the repeatability and long-term beneficial effects of this procedure. If thinning the tissues can create a more suitable visual outcome, then the amount of thickness and the detachment or other associated risks should be justified. Moreover, thinning the tissue can result in unwanted effect of rolling the DEG on itself which can decrease the cell viability. A perfect DMEK/DEG usually has a thickness of 15–30 micrometers which is not enough to hold the lenticule without getting damaged. Therefore, it is very important to understand and validate the mortality threshold for the DEG. This will help to prepare a pre-cut DMEK/DEG in the eye banks and increase the quality control levels, save time, cost, and risks associated with the graft failure due to surgeon preparatory mistakes. Therefore, we believe that a more standardized, validated and ready to transplant tissue should be the future of DMEK surgery. Moreover, the recent advances in the use of FS lasers for donor graft preparation could be advantageous in terms of increasing the stromal interface smoothness and cuting it with more ease; however, it might be more expensive than the conventional techniques. Furthermore, it is also important to validate the procedure for DMEK graft preparation using FS lasers in terms of energy/frequency levels and thickness of the required graft. In the future, use of fibrin glue could prove beneficial for sampling, handling, and transporting the DMEK tissues with ease of surgery, although the pros and cons need to be identified if used in vivo [41]. 

According to a study, if the cost analysis of surgeon-cut and the eye-bank cut donor corneal tissue for EK is valued, then the cost per surgeon-cut donor corneal tissue decreases if the number of cases performed increases per year. Excluding other factors such as opportunity costs, the eye bank processing charge is almost equal to the expenses associated with a surgeon-cut cornea if the surgeon was to perform approximately 15 cases/year. The microfinance study was based on costs of equipments, consumable supplies, labor charges, building space, and risk of attempted damage [42]. Even if the cost is low, the risk associated with the graft preparation during the surgery is of major concern. Therefore, we believe that a prevalidated tissue could be a better option for surgeons to reduce the presurgery time, effort, and risks associated with graft failure.

## 6. Conclusion

Thus, we envision that standardizing the posterior lamellar graft preparation methods will reduce unnecessary manipulation of the tissue in the operating theatre and reduce the high surgical skill or risk quotient. In the near future, the DEG could be supplied as pre-cut tissues which would reduce the overall intervention costs and save time. A recent study has shown a moderate decrease in endothelial cell density if the DEG is left in storage under organ culture, which concludes that a pre-cut DMEK preservation is possible for future transportation [43]. The final graft would reduce the severe efforts of manipulation by the surgeons thus providing better quality tissue for patients. Hence, the intention should be to achieve an easy, efficient, and a validated procedure for DMEK surgery.
